# Treatment of the Supernatant of Anaerobically Digested Organic Fraction of Municipal Solid Waste in a Demo-Scale Mesophilic External Anaerobic Membrane Bioreactor

**DOI:** 10.3389/fbioe.2021.642747

**Published:** 2021-04-12

**Authors:** Antonio Giménez-Lorang, José Ramón Vázquez-Padín, Cecilia Dorado-Barragán, Gloria Sánchez-Santos, Sandra Vila-Armadas, Xavier Flotats-Ripoll

**Affiliations:** ^1^FCC Aqualia, S.A., Madrid, Spain; ^2^Direction of Prevention and Management Services of Área Metropolitana de Barcelona, Barcelona, Spain; ^3^GIRO Joint Research Unit IRTA-UPC, Department of Agrifood Engineering and Biotechnology, Universitat Politècnica de Catalunya UPC-BarcelonaTECH, Barcelona, Spain

**Keywords:** organic fraction of municipal solid waste, anaerobic membrane bioreactor, external membrane, mesophilic, fouling, cross flow velocity

## Abstract

Conventional aerobic biological treatments of digested organic fraction of municipal solid waste (OFMSW) slurries–usually conventional activated sludge or aerobic membrane bioreactor (AeMBR)–are inefficient in terms of energy and economically costly because of the high aeration requirements and the high amount of produced sludge. In this study, the supernatant obtained after the anaerobic digestion of OFMSW was treated in a mesophilic demo-scale anaerobic membrane bioreactor (AnMBR) at cross flow velocities (CFVs) between 1 and 3.5 m⋅s^–1^. The aim was to determine the process performance of the system with an external ultrafiltration unit, in terms of organic matter removal and sludge filterability. In previous anaerobic continuous stirred tank reactor (CSTR) tests, without ultrafiltration, specific gas production between 40 and 83 NL CH_4_⋅kg^–1^ chemical oxygen demand (COD) fed and removals in the range of 10–20% total COD (tCOD) or 59–77% soluble COD (sCOD) were obtained, for organic loading rates (OLR) between 1.7 and 4.4 kg COD⋅m^–3^_reactor_ d^–1^. Data helped to identify a simplified model with the aim of understanding and expressing the process performance. Methane content in biogas was in the range of 74–77% v:v. In the AnMBR configuration, the COD removal has been in the ranges of 15.6–38.5 and 61.3–70.4% for total and sCOD, respectively, with a positive correlation between solids retention time (SRT, ranging from 7.3 to 24.3 days) and tCOD removal. The constant used in the model expressing inhibition, attributable to the high nitrogen content (3.6 ± 1.0 g N-NH_4_^+^⋅L^–1^), indicated that this inhibition decreased when SRT increased, explaining values measured for volatile fatty acids concentration, which decreased when SRT increased and OLR, measured per unit of volatile suspended solids in the reactor, decreased. The alkalinity was high enough to allow a stable process throughout the experiments. Constant CFV operation resulted in excessive fouling and sudden trans-membrane pressure (TMP) increases. Nevertheless, an ultrafiltration regime based on alternation of CFV (20 min with a certain CFV_i_ and then 5 min at CFV_i_ + 1 m⋅s^–1^) allowed the membranes to filter at a flux (standardized at 20°C temperature) ranging from 2.8 to 7.3 L⋅m^–2^⋅h^–1^, over 331 days of operation, even at very high suspended solids concentrations (>30 g total suspended solids⋅L^–1^) in the reactor sludge. This flux range confirms that fouling is the main issue that can limit the spread of AnMBR potential for the studied stream. No clear correlation was found between CFV or SRT vs. fouling rate, in terms of either TMP⋅time^–1^ or permeability⋅time^–1^. As part of the demo-scale study, other operational limitations were observed: irreversible fouling, scaling (in the form of struvite deposition), ragging, and sludging. Because ragging and sludging were also observed in the existing AeMBR, it can be stated that both are attributable to the stream and to the difficulty of removing existing fibers. All the mentioned phenomena could have contributed to the high data dispersion of experimental results.

## Introduction

In spite of being the worst option according to the EU Waste Framework Directive (WFD) prioritization, landfilling is still widely used as a municipal solid waste (MSW) disposal method (24% of MSW and 38.7% of waste in EU according to Eurostat (2018)). This amount can reach up to 100% in some member states, usually in small geographically dispersed populations, where the complexity and cross-effects of logistics for managing the separation and disposal of biowaste at the local level represent a challenge. At best, these populations have to collect the biowaste and send it to centralized biogas and composting plants usually located several kilometers away, whereas in many EU regions (i.e., Romania, Bulgaria), where biowaste or organic fraction of municipal solid waste (OFMSW) is not separately collected, it is sent to landfill or incineration (EC, 2018).

In order to boost the circular economy, resources harvesting from waste are gaining momentum. Opportunities for improving solid waste management are emerging with special focus on the treatment of OFMSW supernatants with the entry into force of the new EU waste directive (EU WFD 2018/851) and the need to separate biowaste at the source before the end of 2023. Anaerobic digestion is the biowaste-to-energy process with the best environmental performance (Ardolino et al., 2020).

Municipal solid waste is mostly composed of organic material that is often treated in anaerobic reactors in waste treatment plants in order to recover energy. In most cases, the obtained digestate undergoes a solid/liquid separation step, producing a liquid fraction (known as anaerobic supernatant) rich in ammonium nitrogen that has to be further treated in order to meet discharge standards (Pedizzi et al., 2018). In Ecoparc 2 (one of the MSW management plants belonging to the Área Metropolitana of Barcelona), OFMSW is processed by dry state anaerobic digestion.

The treatment of leachates and supernatants obtained in solid waste treatment, either in landfills or in source-sorted OFMSW management plants, is challenging from a technical and economical point of view due to high concentrations of contaminants, recalcitrant, and inhibitory compounds (Abuabdou et al., 2020). According to Malamis et al. (2014), the anaerobic supernatant is a liquid stream with specific characteristics that guide the subsequent treatment options; it is characterized by very high N-NH_4_^+^ content and a high chemical oxygen demand (COD)/biochemical oxygen demand (BOD_5_) ratio.

The integration of anaerobic and aerobic processes is gaining increasing interest for the treatment of source-sorted OFMSW. The combined treatment ensures the recovery of energy from the biogas along with the production of compost, which can be used as soil conditioner (Cesaro et al., 2015).

Nowadays, for the treatment of landfill leachate and OFMSW supernatant, one of the technologies used the most is the membrane bioreactor (MBR). The application of aerobic MBR (AeMBR) technology for the treatment of landfill leachate started in the 1990s. Recently Zhang et al. (2020) performed a timely survey characterizing 175 full-scale AeMBRs treating leachate (with individual treatment capacity of ≥100 m^3^⋅d^–1^) in China. The investment and footprint of processes adopting MBRs averaged ∼90,000 CNY⋅m^–3^⋅d^–1^ (∼13,000 USD⋅m^–3^⋅d^–1^) and ∼15 m^2^⋅m^–3^⋅d^–1^, respectively, and the energy consumption was 20–30 kWh⋅m;^–3^ (for a treatment train with MBR + NF/RO).

This high energy requirement need of AeMBR is due to a combination of two factors: first, the high content of COD and N of OFMSW supernatants that need oxygen to be oxidized, e.g., in Ecoparc 2, COD content ranged from 22 to 46 kg⋅m^–3^ and total Kjeldahl nitrogen (TKN) from 5.3 to 6.8 kg⋅m^–3^ according to Pedizzi et al. (2018), Malamis et al. (2014) also reported in their review values ranging from 3 to 80 kg COD⋅^–3^ and from 2.5 to 9.3 kg TKN⋅m^–3^. Second, the high suspended solid content on the mixed liquor of the AeMBR dramatically decreases the oxygen transferability; in order to compensate for this low oxygen transferability, higher air flow is needed (Germain et al., 2007).

With such influent characteristics, cross flow membranes are generally used (Judd, 2011) due to the fact that higher shear stress [cross flow velocities (CFVs) of 1–4 m⋅s^–1^] can be achieved compared with submerged modules (generally lower than 0.25 m⋅s^–1^).

Zuriaga-Agusti et al. (2016) compared the permeate flux obtained from two different OFMSW anaerobic digestion (AD) supernatants: one with low solids [4.4 kg suspended solids (SS)⋅m^–3^, 9.4 kg total COD (tCOD)⋅m^–3^] and a second one with high solids content (12.9 kg SS⋅m^–3^, 32.9 kg tCOD⋅m^–3^). The flux was obtained in AeMBR batch experiments where 40–100 L⋅m^–2^⋅h^–1^ for the first case and only ∼20 LMH for the second one, the more concentrated stream. This low filterability was explained mainly by the higher viscosity, salinity, non-degradable SS concentration, and soluble microbial products (SMP), factors that contributed to a higher fouling rate.

In anaerobic supernatants with high solids content, besides the fouling control requirements in cross flow membranes and aeration requirements, extra energy inputs could be required in AeMBR in terms of cooling energy, since heterotrophic growth is exothermic and the temperature of operation has to be kept under 40°C for stability. Moreover, the ammonia emission to the atmosphere is problematic in aerobic systems, since ammonia stripping was reported as a major ammonia removal mechanism at elevated temperatures with high rate aeration in an open reactor (Visvanathan and Abeynayaka, 2012).

In order to overcome all the above-mentioned limitations of AeMBR, in the present research, anaerobic membrane bioreactor (AnMBR) will be tested for the treatment of anaerobic supernatant.

An AnMBR can be simply defined as a biological treatment process operated without oxygen and using a membrane to provide solid–liquid separation (Lin et al., 2013). Membrane fouling is also the major obstacle to the use of AnMBR. The macromolecules (e.g., proteins, colloids, and bio-refractory pollutants) contained in OFMSW AD supernatant are deposited onto the membrane surface; this phenomenon is exacerbated with increasing strength of the supernatant, which aggravates membrane fouling (Trzcinski and Stuckey, 2009). Membrane fouling is also correlated with the operational control of an AnMBR. When treating landfill leachates, fresh leachate, with higher COD concentrations than intermediate and mature landfill leachates, can lead to higher organic loading rate (OLR) and mixed liquor suspended solids (MLSS) with certain hydraulic retention times (HRTs) of the AnMBR, which tend to aggravate membrane fouling by high production of extracellular polymeric substances (EPS) and SMP (Lei et al., 2018). Besides membrane fouling, it has also been reported that a high OLR, in the range of 13.27–16.32 kg COD⋅m^–3^⋅d^–1^, caused the deterioration of the methanogenesis step and accumulation of volatile fatty acids (VFAs) in an AnMBR (Saddoud and Sayadi, 2007). It has also been claimed that AnMBRs tend to have good performance only at feeding COD < 20 kg⋅m^–3^ or OLR < 10 kg COD⋅m^–3^⋅d^–1^ (Luo et al., 2015).

The use of AnMBR for OFMSW AD supernatant is challenging, since submerged membranes with 4 LMH (Trzcinski and Stuckey, 2010) and very poor fluxes were achieved in cross flow configurations, between 8.3 and 2.5 LMH according to Zayen et al. (2010). However, compared with traditional, granular sludge-based anaerobic treatment technology, AnMBR can overcome the usual features of the anaerobic supernatant, such as high SS, toxicity, high salinity, or drastic changes in OLR (Dereli et al., 2012). Moreover, AnMBR can be combined with further one stage partial nitritation–anammox processes, such as ELAN^®^, where nitrogen will be removed. This kind of systems was successfully implemented in the industrial scale (Vázquez-Padín et al., 2014), and the system requirements fit to the expected features of the produced ultrafiltered effluent: low suspended solids content, low COD/N ratio, and mesophilic temperatures (Pichel et al., 2021). Finally, another significant advantage of AnMBR treating AD supernatants is the favorable energy and environmental balance of AnMBR, which are enhanced in cases of high OLR, efficient membrane scouring, and minimizing dissolved methane (Jiménez Benítez et al., 2020).

Besides filtration performance, biomethane production with AD supernatants from OFMSW is also challenging due to the high ammonium concentration. Westerholm et al. (2011) observed at laboratory scale a 50% reduction in methane production when total ammonia nitrogen (TAN) went up to 5.5 kg⋅m^–3^ caused by ammonia inhibition when treating OFMSW.

The objective of the present study was to assess the operation of an AnMBR treating the liquid fraction of the anaerobic digestate from the OFMSW, in a pilot plant coupled to these facilities and under real field conditions. The final aim is to characterize operational problems and limiting factors to take into account for scaling-up the system.

To the authors’ knowledge, AnMBR technology applied to this kind of waste treatment stream has only been tested at laboratory scale. In terms of technology readiness level (TRL), and specifically understood for this particular application, this corresponds to a TRL = 4. Through this work, we aim to validate the AnMBR technology in a relevant environment, thus increasing the TRL up to 6.

## Materials and Methods

### Analytical Methods

Usual parameters were measured according to standard methods (APHA, 2005): total and volatile solids (TS, VS), total and volatile suspended solids (TSS, VSS), pH, total and partial alkalinity (TA, PA), Kjeldahl and ammonia nitrogen (TKN, N-NH_4_^+^), and tCOD and soluble COD (sCOD). Alkalinity ratio, estimated as (TA–PA)/TA, was used to assess the stability of the anaerobic digestion process, as proposed by Martín-González et al. (2013), and was measured twice a week throughout the pilot plant operation.

Total suspended solids in the reactor were difficult to measure, due to its high concentration at high solids retention time (SRT), and were estimated supposing that TS of the permeate (TS_eff_) approached to total dissolved solids in the reactor. Similar estimation was done for VSS.

Biogas production rate was measured using a displacement column, to which gas was channeled periodically. CH_4_, CO_2_, O_2_, and H_2_S contents were measured by means of a portable gas detector (Dräger, X-am^®^ 7000). VFAs were estimated as acetic acid (AcH) from the intermediate alkalinity measured in permeate.

Anaerobic biodegradability test was performed in triplicate with a sample of the supernatant to be processed, using the purged effluent of the AnMBR as inoculum, following the procedure of Guerrero et al. (1999).

### Influent Processed

The pilot plant was located on the Ecoparc 2 premises (Barcelona, Spain), which processes 80,000 t⋅y^–1^ of OFMSW using dry anaerobic digestion in two reactors, 4,500 m^3^ each. Collected OFMSW from households is pre-treated for the separation of inorganic matter, plastics, and other valuable waste. After anaerobic digestion, digestate follows three serial solid/liquid separation processes, screw press and two centrifugations. The liquid fraction (anaerobic supernatant) obtained is collected in a stirred tank, from where the AnMBR pilot plant is fed after passing through a 1 mm vibrating sieve. Analytical characterization of the influent used throughout the experiments is shown in Table 1.

**TABLE 1:** Characteristics of the anaerobic supernatant used as influent throughout the experiments when reactor was operated as CSTR or as AnMBR.

**Parameter**	**Units**	**CSTR**	**AnMBR**			
			**SRT = 7.1 d**	**SRT = 11.0 d**	**SRT = 17.4 d**	**SRT = 24.0 d**
tCOD	g⋅L^–1^	22.8 ± 1.5	26.8 ± 1.3	27.4 ± 2.1	29.3 ± 3.7	27.1 ± 1.8
sCOD	g⋅L^–1^	13.8 ± 0.9	17.9 ± 0.9	19.8 ± 1.6	18.2 ± 2.8	15.8 ± 1.3
BOD_5_	g⋅L^–1^	5.6 ± 1.3	6.4 ± 0.3	5.9 ± 0.8	3.9 ± 0.6	n.d.
TS	g⋅L^–1^	25.3 ± 0.7	28.2 ± 0.8	27.2 ± 1.3	25.4 ± 0.5	27.3 ± 1.5
VS	g⋅L^–1^	13.7 ± 0.7	16.1 ± 0.8	15.3 ± 1.5	13.6 ± 1.1	14.7 ± 0.8
TSS	g⋅L^–1^	9.6 ± 0.8	9.3 ± 0.6	7.6 ± 1.2	9.6 ± 1.1	11.5 ± 1.1
VSS	g⋅L^–1^	5.2 ± 0.6	5.3 ± 0.5	4.2 ± 0.7	5.1 ± 0.5	6.2 ± 0.6
Total alkalinity	meq⋅L^–1^	326 ± 16	357 ± 4	332 ± 21	340 ± 30	337 ± 20
NH_4_^+^-N	g⋅L^–1^	3.4 ± 0.5	3.7 ± 0.3	3.4 ± 0.3	3.1 ± 0.4	4.0 ± 0.4
TKN	g⋅L^–1^	4.5 ± 0.6	4.5 ± 0.5	4.5 ± 0.4	4.5 ± 0.6	5.1 ± 0.7

*Values are average ± standard deviation (SD). n.d., not determined.*

Regarding the COD content, there is a remaining fraction that corresponds to soluble compounds that are not biodegradable, either by aerobic or by anaerobic microorganisms. This soluble non-biodegradable COD fraction was estimated based on the COD content on the permeate of the existing AeMBR at Ecoparc 2. The weekly average content of this soluble non-biodegradable fraction in 2019 was 1,341 ± 99 mg COD⋅L^–1^ (M.E. Albacete-García, personal communication, 2020).

### AnMBR Reactor

**FIGURE 1:**
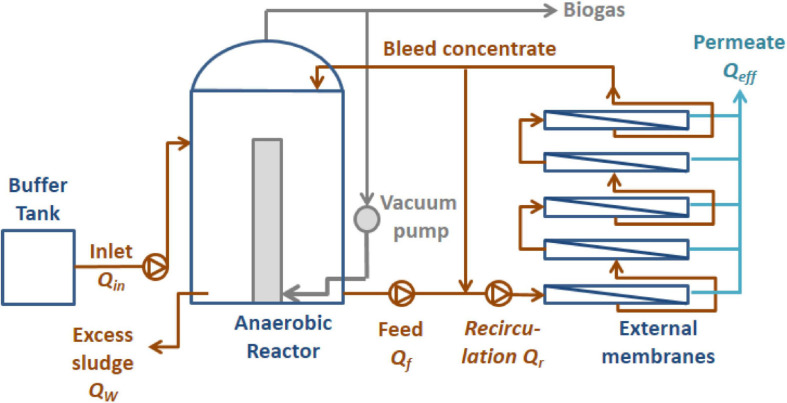
Diagram of the AnMBR reactor and the external ultrafiltration membranes.

The facilities of the AnMBR pilot plant (Figure 1) consist of two main units, the continuous stirred tank reactor (CSTR) and the external ultrafiltration membranes.

The useful volume of the reactor is in the range of 36.8–43.8 m^3^. The reactor is homogenized using a pneumatic stirring system, by recirculating biogas to the bottom of the vessel. Temperature was maintained in the mesophilic range (33–37°C) using an electric boiler and a heat exchanger.

The external ultrafiltration system consists of five serial units of 3 m long tubular membranes of polyvinylidene fluoride (PVDF) with channels 8 mm internal diameter, 30 nm pore size, and total filtration surface 20.5 m^2^ (Berghof Membranes, NL). The system follows the feed and bleed process, by which a pump, flowrate Q_f_, feeds the ultrafiltration system with the reactor effluent and another one, flowrate Q_r_, recirculates the flow in order to maintain the desired CFV in the membranes, between 1 and 4 m⋅s^–1^. Additionally, a third pump drives permeate, flowrate Q_eff_, to an accumulation tank, especially when CFV is low (<2 m⋅s^–1^). The system can be backwashed unit by unit using the permeate, without stopping operation for the rest of the membrane units.

Every 24 h membranes are automatically washed by flushing at low velocity (∼1 m⋅s^–1^). Flushing is also automatically activated when facilities are shut down, in order to avoid sludge accumulation in membranes.

After every experiment, accumulated fibers on membranes were pulled out manually, and a chemical washing (CIP) was done in order to recover permeability, first with a mixture of NaClO (3.5 kg Cl⋅m^–3^) and NaOH (40 kg⋅m^–3^), as proposed by Mohammadi et al. (2003), and then with HCl (36.5 kg⋅m^–3^) for removing mineral precipitates.

The effectiveness of CIP was assessed by measuring the resistance to filtration R after every cleaning process, using tap water and normalizing at 20°C and equation 1. According to Robles et al. (2013), it is assumed that the R value is the sum of intrinsic resistance of membranes (R_M_) and resistance due to irreversible fouling (R_I_), the fouling not removed by the CIP.

(1) $$\text{R}=\frac{\text{TMP⋅}\,10^{8}\cdot 3600}{\eta \,{\text{⋅J}}_{20}},$$

where R is the membrane permeability resistance (m^–1^), TMP is the average trans-membrane pressure for the five membrane units (bar), J_20_ is the standardized flux at 20°C (L⋅m^–2^⋅h^–1^) estimated with equation 2, and η is the viscosity of the permeate (Pa⋅s), estimated with equation 3.

(2) $${\text{J}}_{20}=\text{J}\,\text{⋅}\,1.024^{\left(20-T\right)},$$

where J is the instantaneous flux (L⋅m^–2^⋅h^–1^) measured at the temperature T of the bioreactor effluent (°C).

(3) $$\eta =0.001\,{\text{⋅e}}^{\left(0.580-2.520\,\text{⋅}\,\theta +0.909\,\text{⋅}\,\theta^{2}-0.264\,\text{⋅}\,\theta^{3}\right)},$$

where the dimensionless parameter θ is estimated with equation 4,

(4) $$\theta =3.661\,\text{⋅}\,\frac{\text{T}}{273.1+\text{T}}.$$

The measured *R* value (=R_M_) with new membranes and tap water was 1.38 ⋅ 10^12^ m^–1^.

Permeability of membranes was estimated using equation 5,

(5) $${\text{K}}_{20}=\frac{{\text{J}}_{20}}{\text{TMP}},$$

where K_20_ is the standardized permeability at 20°C (L⋅m^–2^⋅h^–1^⋅bar^–1^).

### Experimental Design

The experiments were designed to test the stability of the anaerobic digestion process, operating the reactor as CSTR without membranes, then to test the efficiency of the AnMBR process, operating at different SRTs and with different membrane CFVs, assessing the global efficiencies of the overall system, and, finally, to report practical difficulties associated to the kind of raw material processed and practical limitations for scaling-up the AnMBR system.

#### CSTR Tests

The bioreactor was operated at five increasing OLR values (Table 2), corresponding to decreasing HRTs, which in this case equals the SRT, at mesophilic temperature (35 ± 0.5°C).

**TABLE 2:** Operational conditions used in the CSTR test.

**OLR (kg tCOD_in_⋅m**^–^**^3^ ⋅d**^–^**^1^)**	**Length (d)**	**HRT (d)**	**tCOD_in_ (g⋅L**^–^**^1^)**	**VS_in_ (g⋅L**^–^**^1^)**
1.73 ± 0.64	21	13.0	23.9 ± 1.8	14.2 ± 0.5
2.19 ± 1.1	11	10.4	23.9 ± 1.1	14.0 ± 0.4
2.83 ± 0.23	11	8.3	21.4 ± 0.8	14.0 ± 0.5
3.38 ± 0.58	13	6.6	22.0 ± 1.2	13.6 ± 0.5
4.43 ± 0.56	10	5.3	23.1 ± 0.7	12.8 ± 0.8

*Average ± standard deviation values.*

The aim was to test start-up and operation under usual OLR values that will be used later in AnMBR experiment and to check whether methane production is consistent with results from the previous biomethane potential test (A), which was 0.270 ± 0.015 NL CH_4_⋅g^–1^ VS_in_ or 0.18 ± 0.01 NL CH_4_⋅g^–1^ tCOD_in_ (Pedizzi et al., 2018).

The reactor was inoculated several weeks before with the same supernatant, since it may content active biomass coming from the previous anaerobic digestion process. The immediate production of biogas and removal of organic matter confirmed that this practice resulted in a good start-up, as in a previous work (Martín-González et al., 2013).

#### AnMBR Tests

The permeability and permeate flowrate values found in the literature when processing leachates or effluents from OFMSW in AnMBR type reactors were very low when compared with other industrial applications. In order to elucidate the relative importance of CFV and the total solids in the reactor, or in the stream to be filtered, on fouling and permeability variations, different operation configurations were used, following an increasing SRT that involves increasing total suspended solids in the reactor (TSS_r_), as indicated in Table 3. For every SRT, different CFV values were also tested, with a maximum value obtained of 3.5 m⋅s^–1^ (CFV_max_), due to the high viscosity of the sludge (Table 3).

**TABLE 3:** Operational conditions utilized in the bioreactor during AnMBR operation.

**SRT [d]**	**HRT [d]**	**OLR [kg tCOD⋅m**^–^**^3^⋅d**^–^**^1;^ kg sCOD⋅m**^–^**^3^⋅d**^–^**^1^]**	**UF test**	**CFV … CFV_max_ [m⋅s**^–^**^1^]**	**Temperature [°C]**	**Length [days]**	**F_v_ [L⋅m**^–^**^2^]**	**TSS_r_ [kg⋅m**^–^**^3^]**	**Average TSS_r_ [kg⋅m**^–^**^3^]**
			CFV2.5 + 1a	2.5…3.5	32.7 ± 1.5	20	3443	14.3 ± 1.1	
Not defined	Not defined	Not defined	CFV2.5 + 1b	2.5…3.5	33.7 ± 1.2	15	2709	20.6 ± 1.1	16.4 ± 3.3

Not defined	Not defined	Not defined	CFV2.5	2.5	34.1 ± 0.3	16	2212	19.7 ± 2.3	19.7 ± 2.3

		5.4 ± 1.6;	CFV2.0 + 1a	2.0…3.0	34.1 ± 0.4	19	3216	19.9 ± 1.9	
7.3	4.8	3.6 ± 1.2	CFV1.5 + 1a	1.5…2.5	32.9 ± 0.5	29	3366	17.6 ± 1.1	18.8 ± 1.8

		4.0 ± 1.1;	CFV1.5 + 1b	1.5…2.5	33.2 ± 0.3	31	3493	17.1 ± 2.5	
11.2	6.8	2.9 ± 0.8	CFV2.0 + 1b	2.0…3.0	37.3 ± 1.7	26	3461	19.5 ± 2.9	18.2 ± 2.7

17.7	10.6	3.3 ± 0.9;	CFV1.0 + 1a	1.0…2.0	33.8 ± 0.8	23	2331	21.9 ± 0.5	21.9 ± 0.5
		2.0 ± 0.6								

24.3	9.3	3.2 ± 1.2;	CFV1.5 + 1c	1.5…2.5	37.7 ± 1.2	41	2983	26.7 ± 2.5	29.6 ± 5.2
		1.5 ± 0.9	CFV1.0 + 1b + BW	1.0…2.0	37.6 ± 1.0	21	2791	34.6 ± 4.2	

*Average ± standard deviation values.*

In preliminary tests, it was observed that operating at constant CFV, fouling formation was fast and it was necessary to stop the system for CIP for a few hours, due to a fast increase in high TMP value (see further discussion about CFV2.5 test). The alternative studied and applied in the current experiments was to alternate cycles of 20 min at a set CFV and afterward 5 min at CFV_max_, which was always the set CFV + 1 m⋅s^–1^, with decreasing CFV_max_ values when TSS_r_ concentration increased (Table 3).

The membrane system was operated with a Q_f_/Q_eff_ ratio higher than 10 at all times, in order to avoid accumulation of solids into the membrane units and to rule out that this was the origin of fouling. All operational conditions were maintained during the time and the accumulated filtered volume (F_v_) of permeate indicated in Table 3.

#### Mathematical Modeling

The continuous variation of operation parameters, such as influent tCOD and flowrate, owing to digestate characteristics variations coming from the large biogas plant, modifies OLR and HRT around the conditions set during experimental planning. This makes it difficult to obtain stationary operating conditions and to explain the results. In order to overcome this limitation, a simple mathematical model has been used, shown in Table 4, in order to have a general framework in which results could be explained and coherence contrasted.

**TABLE 4:** Biochemical coefficients and kinetic rate equations for tCOD and VSS in model 1.

**Process**	**S_b_**	**X_b_**	**X_d_**	**X_I_**	**CH_4_ (Go)**	**Rate**
**Units**	**kg tCOD⋅m^–3^**				**m^3^ CH_4_⋅kg COD_s_^–1^**	**kg tCOD⋅m^–3^⋅d^–1^**
Substrate consumption	−1	Y			(1–Y)⋅0.35	$\frac{\mu_{m}}{Y}\,\frac{S_{b}}{B\cdot X_{b}+S_{b}}\,X_{b}$
Decay of microorganisms		−1	+1			*k*_*d*_⋅*X*_*b*_
Disintegration of X_d_	(1–*f*_*I*_)		−1	*f*_*I*_		*k*_dis_⋅*X*_*d*_

A fraction β of the influent tCOD is considered biodegradable (S_b_), whereas the rest (1–β) of tCOD is non-biodegradable (S_nb_). Furthermore, a fraction α of the influent VSS is considered active biomass X_b_, and the rest (1–α) are organic particles not participating in the biological reactions. The model adopts three processes only, substrate consumption, decay of microorganisms, and disintegration of decayed biomass, in order to avoid a large number of unknown parameters that could not be identified with a relatively low number of experimental values. During the first one, biodegradable substrate S_b_ is consumed by microorganisms X_b_, resulting in new biomass X_b_, contributing to tCOD that cannot be consumed S_nb_, and methane. During the microorganism decay process, the decayed biomass produces an increase of X_d_, whereas the disintegration process produces a fraction *f*_*I*_ that is inert and the remainder (1–*f*_*I*_) is transformed into biodegradable COD S_b_ accessible to bacteria X_b_.

Disintegration of particulate COD and hydrolysis of sCOD are considered the rate limiting steps, since influent is the result of previous anaerobic digestion. In this case, where first order kinetics can be used only when the concentration of microorganisms producing extracellular enzymes is high enough, Contois kinetics can be applied (Vavilin et al., 2008). Contois kinetics has been used in a number of simplified models for expressing the coupled effect of hydrolysis and acidogenesis and, in some cases, methanogenesis, when hydrolysis of particulate organic matter is the rate limiting step of the overall anaerobic digestion process. Vavilin et al. (2004) found that this kinetics is as good at fitting experimental data as the more complex models related to organic particles surface enzymatic reactions, but with less unknown parameters. Owhondah et al. (2016) tested different kinetics combinations for describing the anaerobic digestion of food waste using simplified models with one, two, or three reaction steps. When testing the model with one single reaction, the best experimental data fitting was obtained with the Contois kinetics and also was the result when expressing hydrolysis–acidogenesis when testing the process with two or three reactions. Values for μ_m_ parameter were in the range of 0.136–0.851 d^–1^ and for the B parameter, also called the hydrolysis saturation constant, in the range of 1.4–15.3 kg COD_s_⋅kg COD_b_^–1^. Other authors used this kinetics in simplified models, such as Domenech and Flotats (1997) for predicting the performance of an anaerobic reactor with biomass retention processing the liquid fraction of digested pig slurry under transient loading, Nopharatana et al. (2007) for expressing the hydrolysis of the insoluble fraction of municipal solid waste, Tomei et al. (2008) for the anaerobic digestion of sewage sludge, comparing different substrates to inoculum ratios and after comparing different kinetics, obtaining values of the μ_m_ parameter in the range of 0.136–0.851 d^–1^ and a B value of 1.5 kg COD_s_⋅kg COD_b_^–1^, or Donoso-Bravo and Fdz-Polanco (2010), who adopted higher values for μ_m_ and B parameters for the simulation of different anaerobic reactor configurations for sewage sludge, but including a second reaction using Monod kinetics for expressing the methanogenesis step, the same simplified model structure used by Vavilin et al. (2007), applied to solid materials for studying the effect of mixing intensity.

The Chen and Hashimoto (1980) model is a simplified model using one single reaction step using Contois kinetics, profusely used for expressing the anaerobic digestion of manures, where hydrolysis of organic matter is the rate limiting step. This model is a solution of the model expressed in Table 4 at steady state conditions, with the following simplifications: β = 1, k_d_ = k_*dis*_ = 0. Chen (1983) compiled values from experiments of several authors for the dimensionless product B⋅Y, which was named the K constant of the Chen–Hashimoto model, for the anaerobic digestion of pig manure, concluding that the minimum value was around 0.6 and increasing up to 2, or more, when some inhibition affects the process. Hill (1982) compiled *K* values for other substrates and obtained minimum values around the same of that obtained by Chen (1983) and values up to 10 for poultry manure, for which ammonia inhibition was reported. The parameter B, or the product B⋅Y, could inform whether some inhibition affects the current process if the estimated parameter value is high.

Decay and disintegration processes are expressed with a first order reaction (Batstone et al., 2002). The model is completed with the mass flow in the reactor of X_nb_, S_nb_, and the variables of Table 4, as a function of the HRT. SSV in the effluent is the sum of X_b_, X_d_, X_I_, and X_nb_, whereas tCOD in the effluent is the sum of S_b_, S_nb_, X_b_, X_d_, and X_I_, using 1.42 kg^–1^ COD⋅kg VSS as the ratio for transforming units of VSS related to biomass.

Unknown parameters were estimated fitting the time evolution of experimental values of tCOD and VSS in the reactor effluent and methane production rate throughout the CSTR experiments, minimizing the sum of square differences between the experimental and the estimated values by the model, weighted by the variance of the experimental data for each variable, using the Luus and Jaakola (1973) random direct search method in a region of feasible parameter values. Estimated optimal parameter values were characterized by the Fisher information matrix (FIM), from which the correlation matrix and 95% confidence intervals (CIs) were derived. The Student’s *t* value for analyzing the statistical significance of the parameters in the model was estimated also (Flotats et al., 2003). After obtaining parameter values from fitting experimental data and analyzing the statistical significance of the parameters in the model, model 1 (Table 4) was simplified to model 2, where the disintegration process is not considered (k_dis_ = 0), methane production coefficient (Go) is an independent unknown parameter, and grown biomass is added as new biodegradable substrate.

## Results and Discussion

### Biodegradability Test

The duration of the biodegradability assays was 70 days, the 95% CI of the measured methane potential was 0.112 ± 0.021 Nm^3^ CH_4_⋅kg tCOD^–1^, and the anaerobic biodegradability was 36.8 ± 3.2%. The methane content in the biogas was 73 ± 2%.

### CSTR Tests

During the experiment, the intermediate/partial alkalinity ratio was in the range of 0.11–0.35, and the alkalinity ratio (intermediate/total alkalinity) was in the range of 0.10–0.26, indicating that VFAs are not accumulating and the process was stable, maintaining a pH around 8.3.

**FIGURE 2:**
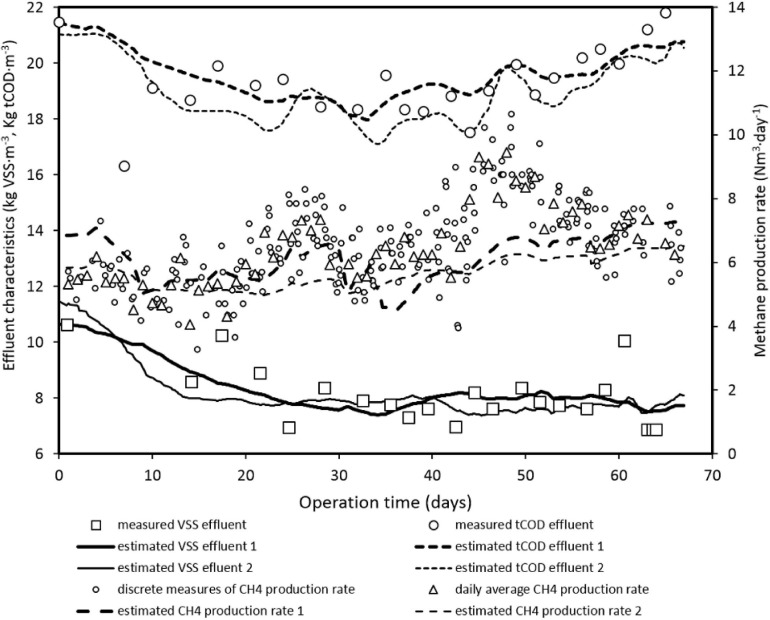
Time evolution of the methane production rate and of VSS and tCOD in the reactor effluent during CSTR operation at the indicated average organics loading rate (OLR) values and the estimated values using model 1 (Table 4) and the simplified model 2. See parameter values in Table 5.

Figure 2 shows the evolution of tCOD, VSS, and the methane production rate throughout the operation of facilities as CSTR, and the estimated values with models 1 and 2. Model 1 (Table 4) allowed good fitting of experimental data, but resulted in parameters that were not significant in the model and, in some cases, without physical or biological sense (see Table 5).

**TABLE 5:** Estimated parameter values for the models applied to CSTR experiments.

			**Model 1**		**Simplified model 2**	
**Parameters**		**Units**	**Mean value ± 95% confidence interval**	***t*-test probability (%)**	**Mean value ± 95% confidence interval**	***t*-test probability (%)**
X_bo_	Initial concentration of active biomass in the reactor	kg COD_b_⋅m^–3^	1.5 ± 0.5	100	8.2 ± 2.5	100
S_bo_	Initial concentration of biodegradable COD in the reactor	kg COD_s_⋅m^–3^	2.82 ± 3.45	91.1	4.0 ± 1.8	100
α	Active biomass in VSS fed	%	0.1 ± 3.6	52.0	27.3 ± 23.7	97.1
β	Biodegradable COD in the feeding substrate@@	%	41.5 ± 11.8	100	37.9 ± 6.8	100
μ_m_	Maximum growth constant	d^–1^	0.33 ± 0.77	76.0	0.16 ± 0.19	94.1
B	Contois kinetics constant	kg COD_s_⋅kg COD_b_^–1^	2.1 ± 11.6	61.8	9.1 ± 13.2	86.0
Y	Biomass yield	kg COD_b_⋅kg COD_s_^–1^	0.32 ± 0.19	99.6	0.16 ± 0.1	98.8
k_d_	Decay constant	d^–1^	0.005 ± 0.190	51.9	0.023 ± 0.016	99.0
Go	Maximum CH_4_ yield	m^3^ CH_4_⋅kg COD_s_^–1^	(1–Y)⋅0.35	−	0.30 ± 0.04	100
k_dis_	Disintegration constant	d^–1^	0.026 ± 10.5	50.2	−	−
f_I_	Non-biodegradable fraction of decayed biomass	Fraction	0.1 ± 270	50	−	−

Probability corresponding to estimated Student’s *t* values, calculated from the inverse of the error covariance matrix of the best linear estimator (FIM), higher than 95% is considered statistically significant in the model. For model 1, this is the case of X_bo_ and Y only, with broad and non-sense 95% CIs for the other parameters and, hence, model 1 cannot explain results, despite the good fitting of experimental values (Figure 2). With the simplification done with model 2, only μ_m_ and B optimal values fail in the statistical significance *t*-test, with broad 95% CIs, including negative values due to the linearity of the estimator, although the correlation coefficient is relatively low (0.74) and the estimated values are of the same order of magnitude of those obtained in simplified models using Contois kinetics (Chen, 1983; Tomei et al., 2008; Owhondah et al., 2016). The rest of the estimated parameters are highly statistically significant, with realistic CIs, although three pairs of parameters present high correlation coefficients: β and S_bo_ (0.97), α and k_d_ (−0.95), and G_o_ and Y (−0.97). These last two parameters were estimated independently but using search regions of realistic and related values. Considering 0.35 Nm^3^ CH_4_⋅kg COD^–1^ for transforming CH_4_ volume units to COD units, the sum of the two estimated parameter values (0.3 ⋅ 0.35^–1^ + 0.16) is 1.02, which is close to the theoretical value of 1, which must be maintained owing to the COD mass balance conservation (Batstone et al., 2002).

The estimated anaerobic biodegradability of the substrate (37.9 ± 6.8%), obtained with the model fitting (Table 5), is consistent with the values measured during the biodegradability test (36.8 ± 3.2%). The estimated Y value obtained is consistent with the sum of average biomass yields for acidogenesis, acetogenesis, and methanogenesis default values used by the ADM1 model (Batstone et al., 2002).

The parameter B, or the product B⋅Y, named K in the Chen and Hashimoto (1980) model, is in the upper range of values found in the literature. The dimensionless product B⋅Y (current optimal estimated value of 1.46) tends to 1 for first order reaction and increases to higher values when substrate concentration and inhibitors, such as ammonia nitrogen, increase (Hill, 1982). The high values obtained for the optimal and the upper range of the product B⋅Y suggest that some inhibition could occur, which could be consistent with the high ammonia concentration measured. Ammonia nitrogen in the reactors was in the range of 2.4–4.3 g NH_4_^+^-N⋅L^–1^, and the estimated free ammonia values were 452–811 NH_3_-N⋅L^–1^, which have been reported as inhibitory in anaerobic digestion processes (Chen et al., 2008; Yenigün and Demirel, 2013), and Pedizzi et al. (2018) obtained better anaerobic digestion performance decreasing the initial ammonia concentration with the same samples used in this experiment. This inhibition can be overcome at high SRT, favoring the hydrogenotrophic methanogenesis pathway (Ruiz-Sánchez et al., 2019).

Using random values within 95% CI model 2 parameter values, considering positive values for μ_m_ and B, and values within 95% CIs for the feeding characteristics (tCOD, VSS), influent flowrate and useful reactor volume used, maximum and minimum methane yields, and tCOD removal were estimated as function of OLR.

**FIGURE 3:**
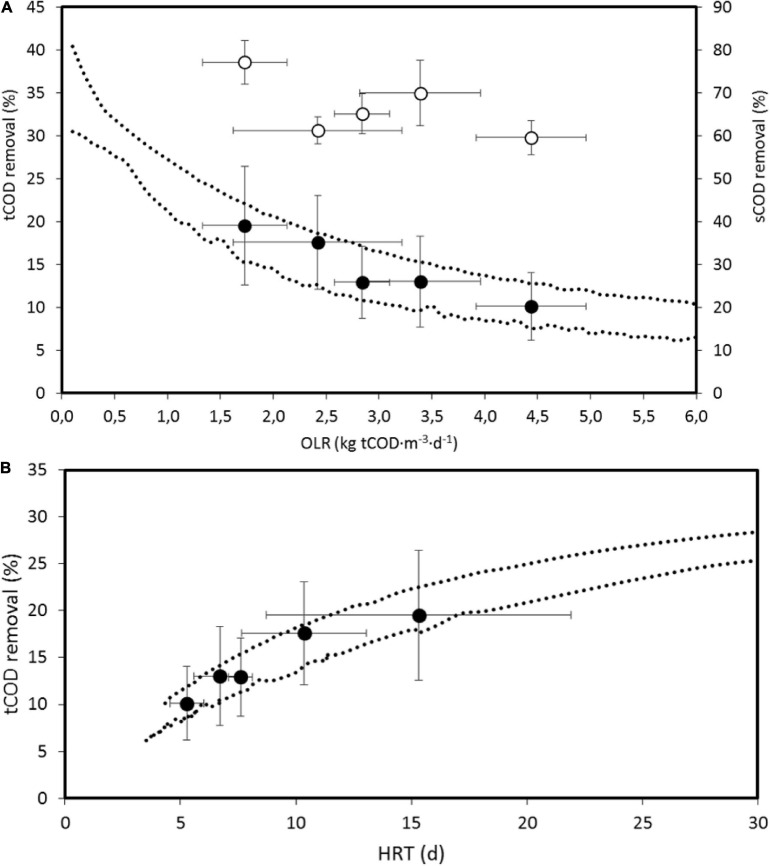
**(A)** Estimated 95% confidence interval (CI) by model 2 of tCOD removal and average experimental values of tCOD and sCOD as a function of the organic loading rate (OLR). Error bars are standard deviations. **(B)** tCOD removal as a function of the hydraulic retention time (HRT), with indication of the 95% CI estimated by model 2. Error bars are standard deviations.

Figure 3A shows the experimental results of the tCOD removal and the expected 95% CI of values when modifying estimated model parameters and experimental conditions within the respective 95% CIs. In this estimation, the variations of flowrate and influent COD and VSS produce wider intervals than the variation of model parameters, which explains the variation of tCOD or sCOD removals, indicated by the standard deviations shown in Figure 3A.

When results are expressed as a function of the HRT, experimental values for tCOD removal are consistent with the estimated CI. The minimum HRT is a value close to 0, due to the continuous inoculation of bacteria through the influent, which allows activity, although low, at low HRT (Figure 3B).

The methane contents of biogas throughout the experiments were in the range of 74–75% by volume, which is high compared with values obtained in the full-scale anaerobic digestion facility processing OFMSW, but consistent with repeated control measures and with the value obtained during the anaerobic biodegradability test.

**FIGURE 4:**
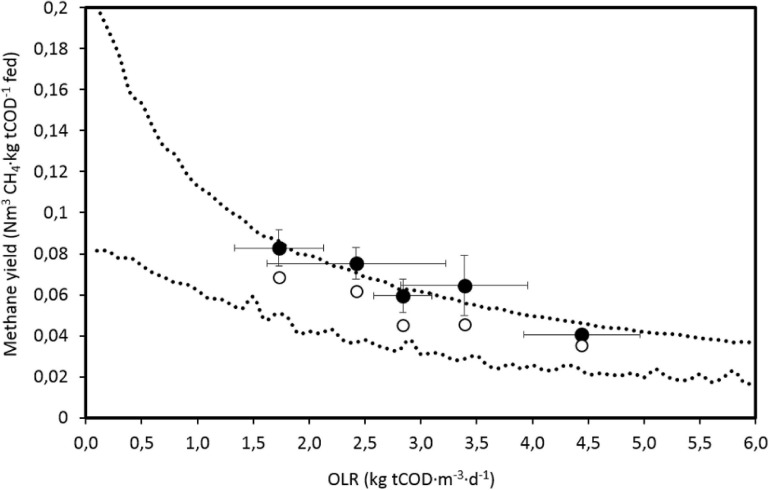
Estimated 95% confidence interval by model 2, measured experimental values of the methane yield and estimated average values based on measured tCOD removal (0.35 Nm^3^ CH_4_⋅kg COD^–1^ removed), as a function of the organic loading rate (OLR). Error bars are standard deviations.

The estimated CIs for the methane yield are consistent with the values from the BMP test done by Pedizzi et al. (2018), 0.18 ± 0.01 Nm^3^ CH_4_⋅kg tCOD^–1^ fed, and from the anaerobic biodegradability test (0.112 ± 0.021 Nm^3^ CH_4_⋅kg tCOD^–1^) fed, corresponding to what can be expected when OLR tends to 0 (Figure 4). The average measured values present a high standard deviation and are close to the upper limit of the estimated CI, and actually, the models tested cannot accurately fit the methane production rate (Figure 2), predicting lower values than those measured. This could be due to an experimental error when measuring gas flowrate or when measuring tCOD, since particulate COD is difficult to determine accurately by standard methods and probably a method adapted to particulate and heterogeneous materials should be used (Noguerol et al., 2012). Nevertheless, the measured values are close to the value suggested by Tchobanoglous et al. (2003), who proposed 0.08 Nm^3^ CH_4_⋅kg^–1^ COD fed for this kind of effluents. The values obtained by Trzcinski and Stuckey (2010) are much higher, in the range of 0.24–0.28 Nm^3^ CH_4_⋅kg^–1^ COD fed, but with an SRT of 30 days and OLR of 11.7 kg COD⋅m^–3^⋅d^–1^.

The measured methane production per unit of tCOD removed for the average values corresponding to each OLR tested is 0.47 ± 0.05 Nm^3^ CH_4_⋅kg^–1^ tCOD removed, which is consistent with values found by Zayen et al. (2010), who obtained yields in the range of 0.37–0.48 Nm^3^ CH_4_⋅kg^–1^ tCOD removed for OLR values between 2.24 and 6.27 kg COD⋅m^–3^⋅d^–1^ in an AnMBR processing landfill leachates. These values are higher than expected, since the tCOD balance of an anaerobic reactor allows a maximum production of 0.35 Nm^3^ CH_4_⋅kg^–1^ COD removed, equivalence used to estimate the methane yield based on the average tCOD removed (Figure 4). When measuring sCOD, the methane yield obtained was 0.15 ± 0.03 Nm^3^ CH_4_⋅kg^–1^ sCOD removed. Considering that part of the sCOD is transformed into microorganisms, the estimated biomass yield is much higher than any biomass yield adopted by the ADM1 model (Batstone et al., 2002), which suggests that sCOD balance cannot be closed without considering solubilization of part of the particulate COD. This is clear from the BMP results of Pedizzi et al. (2018), where COD of the methane produced is higher than sCOD added. This means that a more accurate model should be made fractionating COD and considering more processes and parameters than the simplified model used in this work, which will require more data to be identified.

### AnMBR Tests

#### Biochemical Performance

Suspended matter concentration in the permeate was below the detection threshold (20 mg TSS⋅L^–1^), and therefore, it was considered that the dry matter of the permeate mainly consisted of soluble compounds.

**FIGURE 5:**
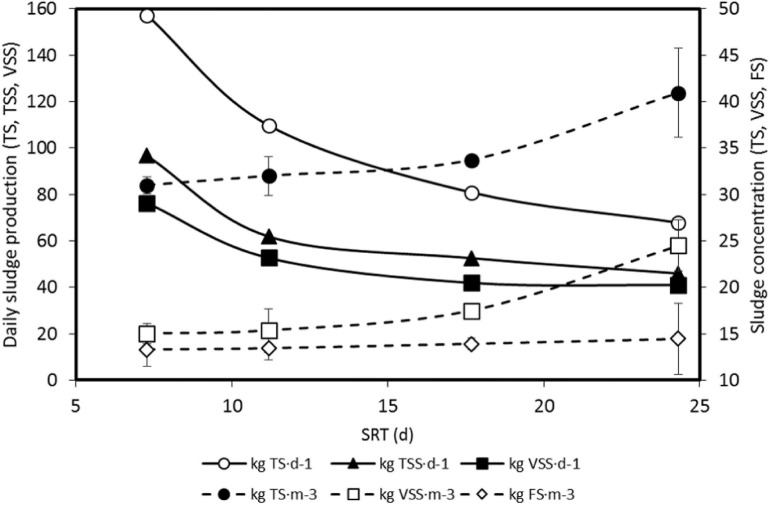
Average daily sludge production in terms of total solids (TS), total suspended solids (TSS), and volatile suspended solids (VSS) and average concentration in the reactor, and in the wasted sludge, of TS, VSS, and fixed solids (FS) for the solids retention time (SRT) values tested. Error bars indicate standard deviation.

With the SRT increase, from 7.3 to 24.3 d, TS in the reactor increased from 31 to 40.9 kg TS⋅m^–3^ and VSS increased from 15.0 to 24.3 kg VSS⋅m^–3^, whereas total fixed solids (FS) increased slightly (Figure 5), indicating that the TS increase was mainly due to organic matter retention (biodegradable and recalcitrant) and biomass growth. Although the increased concentration of suspended matter in the reactor, the average purged daily, or produced daily in the sludge, decreased with the SRT increase, from 157 to 68 kg TS⋅d^–1^ (Figure 5), corroborating the results of Ho and Sung (2009a).

**FIGURE 6:**
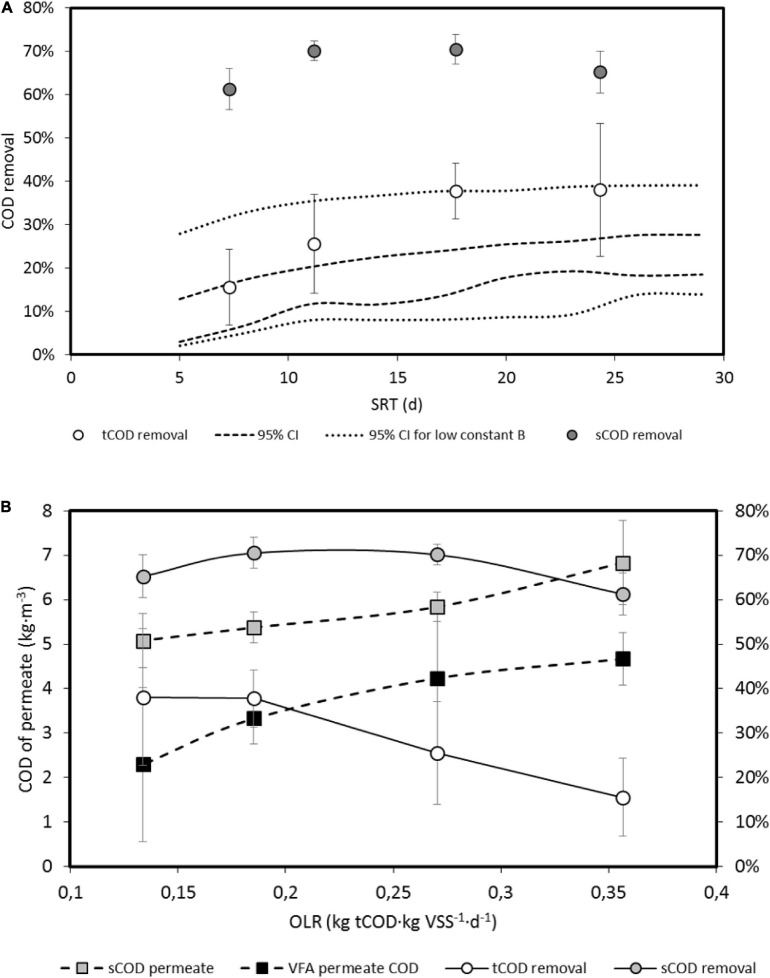
**(A)** Total and soluble COD removals (tCOD and sCOD) for the SRT values tested. Dotted lines express the estimated 95% confidence intervals (CIs) obtained by numerical simulation using model 2 using parameters of Table 5 (95% CI) or using upper interval of parameter B decreased by 1/5. Error bars indicate standard deviation. **(B)** Overall sCOD and tCOD removals and sCOD and VFA concentrations in permeate vs. different OLR. Error bars indicate standard deviation.

To achieve the desired SRT average, the Q_w_ flowrate was defined after measuring the TSS in the reactor value, which was known several days after sampling, delaying the response and making it difficult to maintain a constant given HRT while keeping a permeate flowrate Q_eff_ in the range of 2.1–2.6 m^3^⋅d^–1^. HRT has moved in the range of 4.8–9.3 d, except for 17.7 d SRT, for which HRT was around 11 d (Table 3), obtaining in this situation small variations in almost all parameters measured, as is the case of the concentrations shown in Figure 5, or tCOD removal shown in Figure 6A. Abuabdou et al. (2018) recommended long HRT values for high efficiency AnMBR.

Removal of tCOD was estimated by balancing influents and effluents (permeate and purge) tCOD. Figure 6A shows the experimental values obtained and the estimated 95% CIs when simulating the reactor performance with model 2 and random values into the 95% CI for the kinetic and operational parameters. In order to reproduce the AnMBR configuration, the values adopted for the permeate COD were randomly chosen in the measured range of 19 ± 4% of the purge tCOD. For a given SRT, Q_w_ was defined as the quotient V⋅SRT^–1^, Q_eff_ as the difference between the random Q value (in the range of the experimental values) and Q_w_, and Q_r_ as a random value in the range of 17.5 ± 2.5 times Q_eff_.

For the 95% CI of the kinetic parameters of Table 5, the estimated tCOD interval is the 95% CI indicated in Figure 6A, explaining the measured tCOD removal for 7.3 d SRT only. When the upper limit of the B constant is divided by 5, the 95% CI is wider and can better approach the experimental results. As explained before, an increase of the B constant values, or the product B⋅Y, expresses an increased effect of inhibitors, which seems to be decreased when SRT increases. The higher values of ammonia nitrogen concentration (4.6 g NH_4_^+^-N⋅L^–1^) were measured for 24.3 days SRT, which was also the situation when VFA accumulation was lower.

Figure 6B shows that VFA content in permeate decreases when increasing the SRT or decreasing OLR, expressed as kg tCOD applied per unit of VSS and time, a value that decreases when VSS concentration increases with the SRT increase. The OLR values in Figure 6B correspond to volumetric OLR average values from 3.2 to 5.4 kg tCOD⋅m^–3^⋅d^–1^. Abuabdou et al. (2018) recommended values higher than 2.5 kg tCOD⋅m^–3^⋅d^–1^. The VFA behavior can be understood considering the high ammonia contents of the influent, of the same order of magnitude when bioreactor was operated as CSTR, even higher, but with a lower inhibitory effect when AnMBR is operated at high SRT (low OLR).

Ammonia inhibition has been observed to decrease when operating at high sludge retention time (Bhattacharya and Parkin, 1989) or at HRT higher than 40 d in CSTR (Ruiz-Sánchez et al., 2019), since the acetoclastic methanogenic pathway shifts to the hydrogenotrophic pathway by the action of syntrophic acetate-oxidizing bacteria (SAOB), which are more tolerant to high ammonia levels than acetoclastic methanogens (Wang et al., 2015). Since SAOB doubling time is much higher than acetoclastic methanogens, high SRTs are needed to avoid SAOB washout (Westerholm et al., 2016), and Tian et al. (2018) obtained a successful acclimation at high ammonia nitrogen concentration with an HRT of 23 days. This suggests the presence of SAOB when increasing the SRT on the AnMBR tests and favoring VFA consumption.

Moreover, the measured average tCOD removal values at high SRT (around 38%) are on the upper limit of the wider estimated 95% CI, a value close to the maximum biodegradability minus the minimum biomass yield. The estimated CH_4_ yield from the average experimental tCOD removals is higher than the upper limit of the estimated 95% CI for low B values and for SRT values higher than 17 d (data not shown). The observation made by Trzcinski and Stuckey (2010) of a high degradation of recalcitrant compounds in an AnMBR at high SRT, fed with an effluent similar to that of the present study, could explain the current results of tCOD removals, higher than the measured and estimated biodegradability, suggesting a positive effect of the AnMBR operating parameters over the recalcitrant organic matter.

Figure 6A shows the sCOD removal, estimated from the sCOD overall balance, obtaining average removals up to 70%. The fraction of the effluent tCOD exiting the system in the permeate flowrate varied from an average of 11% for low SRT to 21% for high SRT, with permeate sCOD values indicated in Figure 6A. Soluble sCOD not considering VFA for high SRT (24.3 d) or low OLR (0.13 kg tCOD⋅kg VSS^–1^⋅d^–1^), see Figure 6B, is 54.7% of total permeate sCOD, whereas this percentage is 31.6% at low SRT, high OLR. Despite the overall tCOD removal, this indicates a probable higher hydrolysis rates of particulate organic matter at high SRT, explaining the decrease of the sCOD removal as well and confirming the results of Pedizzi et al. (2018) and Albacete-García (2017).

As has been shown in Figure 5, increasing the SRT entails decreasing the excess biomass purge and therefore increasing the suspended matter in the reactor, which is usually associated with higher fouling rates, both in AnMBR (Ho and Sung, 2009b; Lin et al., 2013) and in AeMBR (Meng et al., 2009). Moreover, in case of increasing SRT above the tested values, an accumulation of inert matter in the reactor could decrease the specific biomass activity (Ahmed and Lan, 2012). Finally, high TSS content in the reactor makes the system more sensitive to CFV deviations and increases the possibility of the “sludging” phenomena.

Since there is a clear positive correlation between TSS_r_ and apparent viscosity (Henkel, 2010), an increase on TSS_r_ involves an increase in the energy consumption required to maintain the cross-flow speeds that are required for membrane cleaning. Furthermore, high apparent viscosities cause unwanted temperature increases due to the friction generated during the recirculation process. This is clearly evident in the test CFV1.0 + 1 + BW, where the temperature reached an average of 37.6 ± 1.0°C (Table 3). This suggests that the TSS_r_ content has a strong influence on the temperature increases in sidestream MBR, even at the lowest tested CFV (1 m⋅s^–1^). In the test CFV1.5 + 1c, a high temperature was also observed despite operating at a lower TSS_r_ content than in CFV1.0 + 1 + BW, which suggests that both CFV and TSS_r_ have a relevant influence on the increase in temperature. This is undoubtedly a limitation for the process itself, since the increase in temperature does not reach an equilibrium and therefore can exceed critical temperatures for anaerobic biomass.

The pH values were very stable during all the analyzed periods (minimum pH was 8.08 ± 0.06 in test SRT = 7.3 and maximum pH was 8.12 ± 0.07 in SRT = 24.3), which can be attributed to the high alkalinity within the bioreactor. Agreeing with other references about AnMBR treating young leachates or wastewaters of similar composition (Zayen et al., 2010; Martín-González et al., 2013), pH of the reactor is not a good indicator of acidification. The value considered critical (IA/PA = 0.3, according to the method proposed by Martín-González et al., 2013) has never been exceeded, which concludes that the VFAs were maintained in such a range that indicates a balanced anaerobic biological process.

In order to maximize COD removal in the AnMBR, further research should be aimed at two strategies, both linked to reducing the inhibitory effect of ammonia on methanogenic biomass: (1) remove suspended matter in the influent anaerobic supernatant in order to grow more biomass inside the reactor, instead of accumulating non-biodegradable organic matter, while increasing the SRT and consolidating adapted biomass (SAOB) to ensure the process stability and (2) remove ammonia in the inlet water through pre-treatments, such as stripping, in order to reduce the inhibition (Pedizzi et al., 2018).

#### Permeability and Membrane Ultrafiltration Performance

The AnMBR was operated at J_20_ between 2.8 and 7.3 L⋅m^–2^⋅h^–1^ and at K_20_ between 3.8 and 0.9 L⋅m^–2^⋅h^–1^, respectively. Apparently the flux stabilizes in the range of 3–4.5 L⋅m^–2^⋅h^–1^ after the first ultrafiltered 18 m^3^⋅m^–2^. These values are of the same order of magnitude as those reported in AnMBR with similar effluents (Trzcinski and Stuckey, 2010; Zayen et al., 2010; Cirik and Gocer, 2020), but significantly lower than other sidestream mesophilic AnMBR treating industrial effluents (Dereli et al., 2012). It is important to note that many of the mentioned published results may not always be clearly expressed at standard conditions (i.e., temperature = 20°C).

On the one side, analyzing the sludge filterability (expressed as additional resistance measured after a volume of 20 L⋅m^–2^_membrane_ was ultrafiltered in cross flow conditions) of several AnMBR treating industrial effluents, Odriozola et al. (2021) obtained the worst sludge filterability in the anaerobic sludge of our prototype. According to this study, the dosage of flux enhancer for increasing sludge filterability was significantly higher than any other studied industrial effluent, probably because of the high amount of SMP, which caused a decrease in availability of the flux enhancer.

On the other side, fluxes obtained in the current work are sustained over time, registering a total of 241 days of active filtration (Table 3). When expressing the length of ultrafiltration in terms of ultrafiltered volume, up to 30 m^3^⋅m^–2^ have been achieved. Adding individual tests, not shown, the time length increased to 331 days and 42 m^3^⋅m^–2^, respectively.

In some of the previous experiences with AnMBR treating landfill leachate, low permeability, or even membrane collapse (Trzcinski and Stuckey, 2010; Do, 2011), could be explained because of the use of a submerged membrane configuration, where applied CFV used to be significantly lower (<0.25 m⋅s^–1^) than external membrane configuration. These low CFVs could not be enough to remove an excessive cake layer. In the case of Zayen et al. (2010), where the conditions were very similar to our case (a sidestream mesophilic AnMBR operating at CFV = 3 m⋅s^–1^ and treating a young landfill leachate with similar composition), TSS_r_ was <3 g⋅L^–1^, a concentration far out of the usual range for optimal operation in MBR technology, which could explain the low fluxes obtained and the sharp flux decreased in only a few days.

It could be stated that the length of our experience can be attributed to the CIP done after each test (after ultrafiltrating approximately 2.2–3.5 m^3^⋅m^–2^ or after 15–41 consecutive operation days; see Table 3). However, CIP is a useful mechanism for cleaning fouled membranes, but not for improving low permeability or non-optimal operating conditions.

Two operational parameters were considered to influence the permeability loss: SRT and CFV.

Since contradictory conclusions have been reported, the effect of SRT influence on membrane filtration performance requires further research (Meng et al., 2009; Dereli et al., 2012). High SRT implies a high sludge concentration that has shown to negatively affect the critical flux in submerged AnMBR and to decrease the flux rates (Jeison and van Lier, 2006). A reliable explanation to this is the pronounced cake compaction due to rapid cake layer build up (Dereli et al., 2012). On the other hand, the lower the SRT, the generally lower the removal of sCOD (as shown in Figure 6A), including the removal of SMP, which are usually considered one of the main biofoulants (Du et al., 2020).

The influence of CFV on the permeability K_20_ can be either positive or negative. On the one hand, it was reported that by increasing CFV, the resistance due to the cake layer could be decreased, as long as the Reynold’s number was below 2,000 (Choo et al., 2000) and to some extent put membrane fouling under control (Hai et al., 2014). Therefore, the relative permeability loss is expected to be close to 0 when increasing CFV.

On the other hand, it is well known that higher CFVs are associated with breakdown of microbial flocs leading to smaller size of sludge flocs in AnMBR, to a higher concentration of soluble cell products (SMP), and, finally, to an increase of the cake layer resistance (Jeison and van Lier, 2006; Ho and Sung, 2009b). High CFVs are also related to the reduction of the cake layer thickness, which acts like a barrier against the fine particles, thus increasing the internal fouling of the membrane (Choo and Lee, 1998).

**FIGURE 7:**
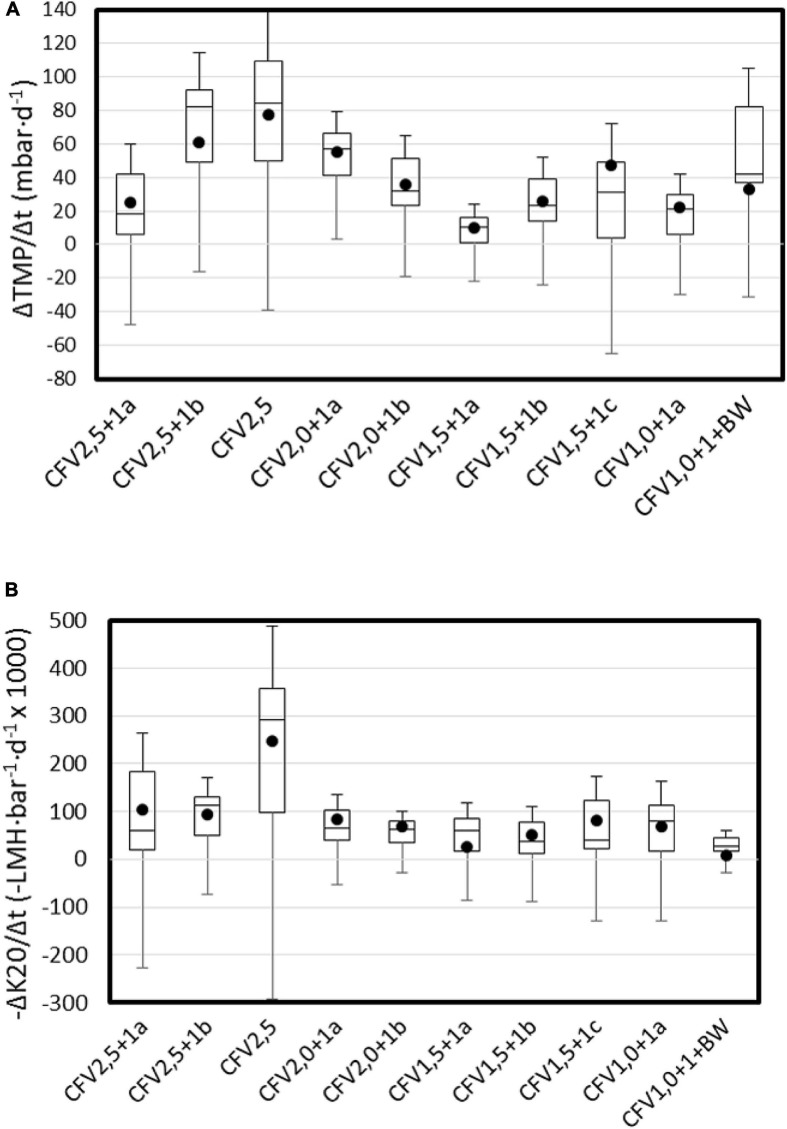
Box plot for **(A)** daily TMP increase and **(B)** daily permeability loss.

In an attempt to quantify the loss of permeability, two daily rates were proposed: daily TMP gradient (ΔTMP/Δt) and daily K_20_ gradient (ΔK_20_/Δt). In the case of K_20_, the absolute value for each period was represented, meaning that the higher the value, the higher the K_20_ loss. In both cases, negative values mean fouling removal, i.e., improvement of ultrafiltration performance. Figure 7 represents both parameters, ordered from the higher CFV to the lowest CFV.

Unlike other papers, tests were driven using alternation of CFV, as described in the Materials and Methods section. The only test using a constant CFV equal to 2.5 m⋅s^–1^ (test CFV2.5) stopped due to excessive TMP after only ultrafiltrating 2,212 L⋅m^–2^ (Table 3). This test also recorded the highest relative loss of permeability (up to 0.247 LMH⋅bar^–1^⋅d^–1^) (Figure 7). Before and after this test, other attempts at constant CFV (even up to 3.5 m⋅s^–1^) were made, but in all the cases, the TMP raised rapidly (results not shown) after a few hundred ultrafiltered L⋅m^–2^. Thus, alternating CFV could be the key to controlling the cake layer growth and maintaining subcritical conditions and, thus, warrant a sustainable long-term UF process without shutdowns due to excessive TMP values.

Except for the CFV2.5 test, average permeability losses were usually in the range of 0–0.1 LMH⋅bar^–1^⋅d^–1^, and no clear trend seems to exist between CFV and TMP or permeability loss. Trzcinski and Stuckey (2010) reported membrane collapse in a submerged AnMBR treating leachate coming from hydrolyzed OFMSW operating at mesophilic range and at SRT = 30 days, whereas no membrane collapse was observed operating in the same conditions but at SRT = 300 days. In this study, no clear trend seems to exist between SRT and TMP or permeability loss. It is likely that other factors (besides CFV and SRT) impact permeability loss, which confirms the usual statement that fouling process on MBR technology is a complex process that is dependent on many factors and cannot be explained by a single factor.

An interesting phenomena is the low permeability loss observed on the CFV1.0 + 1b + BW experiment, which may have been a consequence of the automatic backwash routines. The low value obtained (0.009 L⋅m^–2^⋅h^–1^⋅bar^–1^⋅d^–1^) indicates that permeability was constant throughout all the experiments, even at extreme high TSS_r_ contents.

**FIGURE 8:**
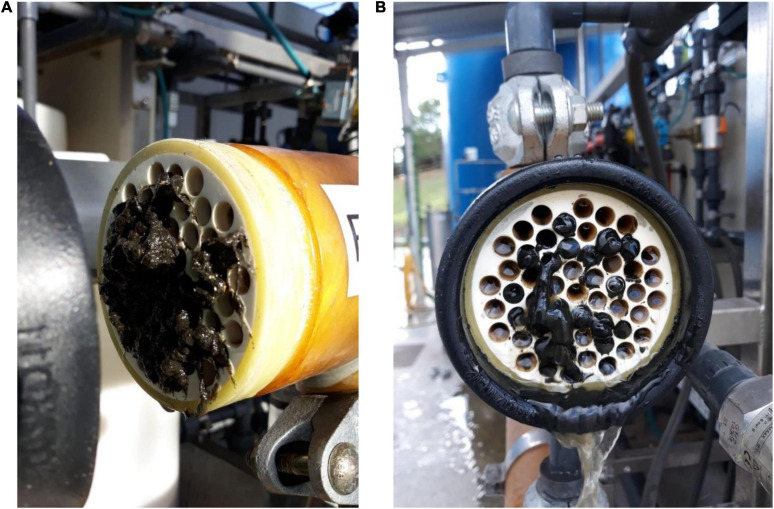
Ragging **(A)** and sludging **(B)** observed on the external ultrafiltration membranes.

Permeability loss could also be influenced by clogging phenomena that were often observed: ragging (accumulation of macroscopic fibers at the entrance of the modules) and sludging (accumulation of highly concentrated suspended matter in the channels of the membranes) (Figures 8A,B). The ragging phenomenon occurred despite the existence of a self-cleaning filter with a 1 mm mesh size located at the entrance of the tubular membranes. The problem with ragging in tubular membranes is that, as fibrous materials accumulate in the cross-flow circuits, the pressure drop in the circuit begins to increase. As a consequence, the recirculation pump increases its rotation speed (Hz) to reach the Q_r_ setpoint, which brings about an exponential increase in the energy consumption of the treatment, reducing the efficiency of the treatment and its potential benefits compared with aerobic alternatives. Furthermore, by plugging the inlet to the membranes, it causes the ultrafiltration not to be distributed homogeneously over the entire membrane surface. If the head loss is such that the pump is unable to reach the setpoint CFV, then the sludge circulating between the membranes gets concentrated as permeate is being extracted, thus increasing the risk of sludging. In keeping with Gabarrón et al. (2013), the only option for unclogging was manual removal of both the intercalated fibers and the accumulated sludge. In the case of sludging, it was necessary to unclog channel by channel using pressurized water. We can state that after removing the fibers accumulated at the entrance of the membranes, the K_20_ values improved significantly.

**FIGURE 9:**
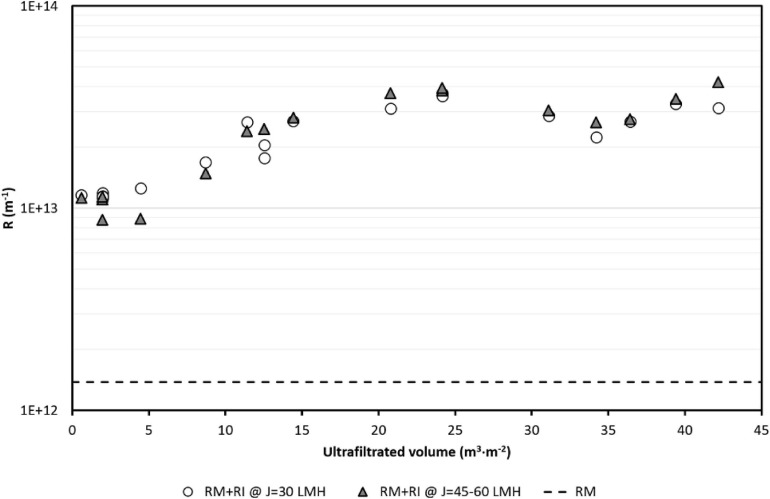
Evolution of filtration resistance (R_M_ + R_I_) measured after CIP cleaning.

A remarkable increase in resistance attributable to irreversible fouling (R_I_) was observed in the first filtered 15 m^3^⋅m^–2^. Afterward, and up to 42 m^3^⋅m^–2^, irreversible fouling seems to remain constant (Figure 9).

Albacete-García (2017) also observed irreversible fouling in commercial membranes through dead-end filtration tests in the same anaerobic supernatant used here. Zhang et al. (2007) also reported irreversible resistance due to inorganic precipitation in a side stream AnMBR treating swine manure at CFV of 2 m⋅s^–1^, even with frequent CIP. The increase values obtained in R_I_ were significantly lower (1.3 times) than those obtained in these experiments (about one order of magnitude more). Although we tried to be as rigorous as possible, the R_M_ + R_I_ estimates were done just to provide some idea about irreversible fouling, assuming that the demo conditions performed in this study are less exact than laboratory conditions; for instance, despite several flushings, in the tap water used for quantifying R_M_ + R_I_, turbidity could still be observed, which probably negatively affected the results.

**FIGURE 10:**
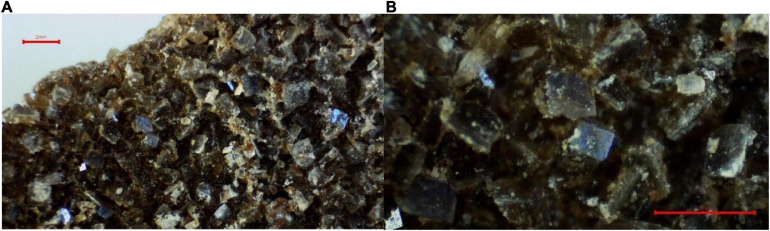
Photographs of scanning electron microscopy (FEI-Quanta 200) under vacuum conditions and EDS system. Struvite crystals in **(A)** a millimeter scale and calcium precipitates in **(B)** a micrometer scale.

In any case, in the AnMBR prototype, the formation of mineral precipitates in pipes was constant, especially in smaller diameter pipes, and in sections where the sectional velocity was low. Scanning electron microscopy identified the main mineral precipitates, which were struvite (MgNH_4_PO_4_ ⋅ 6H_2_O) and calcium oxide (Figure 10). Struvite is a compound considered by the literature as one of the main causes of inorganic fouling in AnMBR (Lin et al., 2013). Cirik and Gocer (2020) also observed calcium precipitate in an AnMBR treating leachate. Finally, higher SRTs in AnMBR were related to higher inorganic fouling (Kang et al., 2002). Cleaning with diluted HCl could dissolve the precipitates in pipes. Nevertheless, as shown in Figure 9, HCl CIP could not avoid irreversible fouling.

Hence, future research should be aimed at testing cleaning reagents (including ingredients such as chelators, detergents, etc.) that allow maximum removal of foulants, that is, maximize the recovery of the membrane’s filtration capacity and decrease as much as possible the R_I_ values. The obtained values (R_M_ + R_I_) using tap water have been similar, regardless of the operated flux.

Ragging and the associated sludging risk, as well as inorganic and irreversible fouling, occurred frequently in the AnMBR test and significantly affected the performance of the filtration system. Further research should be focused on finding solutions and strategies that help to overcome the mentioned drawbacks, from testing cleaning reagents that allow maximum removal of foulants to new options to remove fibers.

## Conclusion

According to the anaerobic biodegradability assays, the anaerobic supernatant coming from OFMSW digestion operating at mesophilic range can be degraded up to 36.8 ± 3.2% tCOD, having a potential of generating up to 0.112 ± 0.021 Nm^3^ CH_4_⋅kg tCOD^–1^. In a demonstration scale AnMBR (40 m^3^ of anaerobic reactor volume and 20.5 m^2^ of cross flow membrane surface), the OLR applied ranged from 1.5 to 5.4 kg COD⋅m^–3^⋅d^–1^, whereas sCOD removal ranged from 61 to 70%.

The UF membranes operated up to 331 days and up to 42 m^3^⋅m^–2^, respectively, being the flux and the K_20_ range 2.8–7.3 L⋅m^–2^⋅h^–1^ and 0.9–3.8 L⋅m^–2^⋅h^–1^⋅bar^–1^, respectively. Despite the poor filterability of the sludge reported in recent references, extreme operations, such as TSS_r_ content up to 34.6 ± 4.2 kg⋅m^–3^, did not lead to membrane collapse or excessive fouling. Instead of operating at constant CFV, alternating CFV proved to be the key to controlling cake layer growth and maintaining subcritical conditions and, thus, warrants a sustainable long-term UF process.

A simplified dynamic model was built, and most of the estimated parameters are highly significant, including the anaerobic biodegradability of the substrate, explaining the reactor performance. The model suggests the presence of inhibition due to ammonia nitrogen. The model and the experimental data demonstrate that the inhibitory effect of ammonia decreases at high SRT or low OLR.

Hence, in order to maximize COD removal in the AnMBR, further research should be focused on two strategies, both linked to the reduction of the inhibitory effect of ammonia on methanogenic biomass: remove suspended matter in the influent anaerobic supernatant in order to increase the SRT or remove ammonia in the inlet wastewater by complementary pre-treatments.

## Data Availability Statement

The raw data supporting the conclusions of this article will be made available by the authors, without undue reservation.

## Author Contributions

AG-L compiled most of the results and drafted the manuscript, except introduction. JV-P drafted the introduction, designed the experiments, and participated actively in the interpretation of results. CD-B carried out majority of the experiments with CSTR and AnMBR. GS-S and SV-A coordinated the analytical tasks, supervised the study, and set up the manuscript formatting guidelines. XF-R carried out the biodegradability test, the modeling and the simulation tasks, as well as the interpretation of results. All authors read, corrected, and approved the final manuscript.

## Conflict of Interest

AG-L, JV-P, and CD-B were employed by the company FCC Aqualia, S.A. The remaining authors declare that the research was conducted in the absence of any commercial or financial relationships that could be construed as a potential conflict of interest.
